# Exploring the Interplay Between Molecular Signaling and Ecosystem Resilience in Plants Under Salt and Water Stress

**DOI:** 10.3390/plants15020229

**Published:** 2026-01-12

**Authors:** Alexandre Maniçoba da Rosa Ferraz Jardim, Toshik Iarley da Silva

**Affiliations:** 1 Department of Biodiversity, Institute of Biosciences, São Paulo State University—UNESP, Rio Claro 13506-900, SP, Brazil; 2 Center for Agrarian, Environmental, and Biological Sciences, Universidade Federal do Recôncavo da Bahia—UFRB, Cruz das Almas 44380-000, BA, Brazil

## 1. Introduction

Plants represent the cornerstone of terrestrial ecosystems and the foundation of global food security, playing a pivotal role in achieving the United Nations’ Sustainable Development Goal 2 (Zero Hunger). Nevertheless, the 2024 Sustainable Development Goals Report highlights a critical scenario in which progress has stagnated or even reversed, with approximately 8.2% of the global population currently facing chronic hunger [1]. This situation is further exacerbated by armed conflicts and an intensifying climate crisis, which have increased the frequency and severity of drought events and accelerated soil salinization worldwide.

Drought and salinity are among the most pervasive constraints on plant productivity, and although they originate from distinct environmental drivers, they converge on a shared physiological core dominated by osmotic stress and redox imbalance. Water deficit directly lowers soil water potential, while salinity reduces the osmotic potential of the soil solution, often imposing a “physiological drought” even when moisture is present. In both cases, impaired water uptake triggers rapid stomatal limitation (frequently mediated by abscisic acid) thereby restricting CO_2_ assimilation and predisposing the photosynthetic apparatus to excess excitation energy and electron leakage, which enhances the formation of reactive oxygen species (ROS) in chloroplasts and mitochondria. When ROS production overwhelms antioxidant capacity, oxidative damage ensues, including lipid peroxidation, protein oxidation, and membrane destabilization, aggravating cellular dehydration and loss of compartmentalization. Salinity further adds an ionic component (Na^+^ and Cl^−^ accumulation and K^+^ and Ca^2+^ disequilibrium) that intensifies metabolic disruption and accelerates oxidative and osmotic injury, underscoring the tight mechanistic coupling between drought- and salt-induced stress syndromes in plants.

Plant responses to these stresses are not linear but are mediated by a complex network of molecular signaling pathways that translate environmental perception into physiological adaptation. Recent advances in translational research, moving from model species such as *Arabidopsis thaliana* to major crop plants, have highlighted both the conservation and divergence of regulatory pathways. For instance, Mitogen-Activated Protein Kinase (MAPK) signaling may exert opposite effects on the stability of transcription factors such as ICE1 across different species, thereby complicating straightforward genetic engineering approaches [2]. Hormonal coordination plays a central role in these responses, with abscisic acid (ABA) acting not only in stomatal closure but also engaging in extensive crosstalk with ethylene and brassinosteroids to modulate root growth and senescence under stress conditions. In addition, calcium (Ca^2+^) signaling and activation of the Salt Overly Sensitive (SOS) pathway remain key paradigms for sodium (Na^+^) extrusion and the maintenance of ionic homeostasis, ultimately enabling cellular survival in saline environments [3].

To mitigate oxidative and osmotic damage, plants deploy sophisticated metabolic adjustments and molecular “memory” mechanisms. The exogenous application of priming agents, such as melatonin and *N*-acetylglutamic acid (NAG), has been shown to reprogram gene expression and enhance the activity of antioxidant enzymes even prior to the onset of severe stress [4]. Recent studies indicate that such chemical priming can induce epigenetic modifications, including histone acetylation, which facilitate the rapid transcription of stress-responsive genes, thereby conferring robust and, in some cases, transgenerational tolerance [5]. At the metabolic level, these responses translate into the accumulation of compatible osmolytes (e.g., proline and soluble sugars) and membrane lipid remodeling, which are essential strategies for stabilizing the cellular machinery against dehydration and ionic toxicity [6].

At the agronomic frontier, the translation of these physiological concepts to field conditions has been successfully achieved through management strategies such as Regulated Deficit Irrigation (RDI). In crops such as grapevine (*Vitis vinifera*), RDI not only promotes water savings but also improves fruit quality by concentrating secondary metabolites and soluble sugars, thereby balancing excessive vegetative growth with reproductive development. This approach, which exploits the plant’s natural hormonal signaling, particularly root-to-shoot abscisic acid signaling, to regulate transpiration, represents a key tool for agricultural sustainability in arid and semi-arid regions [7].

## 2. Overview of the Special Issue

**Figure 1:**
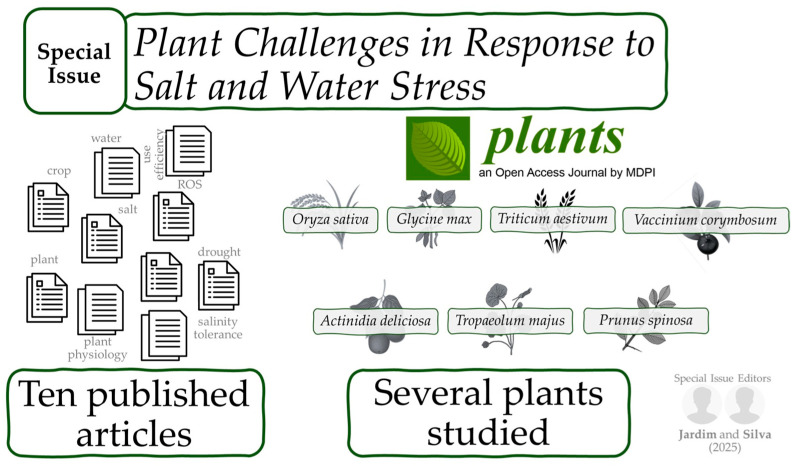
Overview and most published topics in our Special Edition.

With the fast-growing demand for resilient crops in arid and semi-arid regions, it is crucial to understand the cross-scale interactions that govern stress tolerance. In our Special Issue on “Plant Challenges in Response to Salt and Water Stress”, ten papers were included to address the molecular mechanisms, physiological responses, and ecosystem dynamics associated with abiotic stress (Figure 1).

Herein, the ability of plants to adjust molecular signaling for survival is reported for model species like *Arabidopsis* [8] and major crops like rice (*Oryza sativa*) [9]. The published studies also report the physiological and metabolic costs of stress in soybean (*Glycine max*) [10] and the potential of genetic resources in ancient wheat (*Triticum aestivum*) [11]. Furthermore, these studies highlight the role of mitigation strategies, such as chemical priming in nasturtium (*Tropaeolum majus*) [12], soil amendments in blueberry (*Vaccinium corymbosum*) [13], and regulated deficit irrigation in kiwi (*Actinidia deliciosa*) [14]. Expanding the scope, the ecological roles of non-crop species, such as *Prunus spinosa* [15] and moss crusts [16], alongside vegetation structure [17], were examined to understand water conservation at the landscape level (Figure 1).

The lessons learned from these studies include, but are not limited to, the following: (1) Molecular gatekeepers: specific signaling pathways, such as CPK2-mediated ABA signaling [8] and γ-aminobutyric acid (GABA) accumulation [9], act as primary gatekeepers for ion homeostasis and root adaptation. (2) Metabolic coupling: Stress responses such as growth and photosynthesis are often coupled with metabolic shifts. Falcioni et al. [10] demonstrated that progressive water deficit creates a specific metabolic footprint that precedes morphological damage. (3) Management as a modulator: mitigation strategies can uncouple stress from yield loss. Priming with salicylic acid cocktails [12] or using fulvic acid [13] acts as a “modulator”, enhancing the plant’s internal defense systems before severe damage occurs. (4) Ecological buffering: Resilience is not solely a plant trait but an ecosystem service. Mosses [16] and understory vegetation [17] function as hydraulic buffers, modifying the micro-environment to favor survival.

## 3. The Complex System of Stress Resilience

The dynamics of interaction within the soil–plant–atmosphere continuum (SPAC), when subjected to abiotic stress conditions, trigger a complex reorganization of cropping systems that goes beyond a simple reduction in growth. These disturbances elicit integrated responses across multiple biological scales: “structural” changes, evidenced by phenotypic plasticity in root system architecture, whereby the plant modulates lateral root growth and root density to optimize soil exploration [18]; “chemical” changes, mediated by the systemic signaling of phytohormones such as abscisic acid and by ionic redistribution processes [19]; and “biological” changes, marked by the fine regulation of the enzymatic antioxidant system to mitigate the toxicity of reactive oxygen species (ROS) [20]. Plant resilience, therefore, emerges as the net product of cellular homeostasis maintained under these altered systemic states.

Challenging the traditional view that stress is invariably deleterious, the agronomic value of carefully managed, moderate stress has been recognized for decades, and more recent literature has helped formalize this long-standing principle under the “eustress” framework within agronomic management. Strategies such as RDI demonstrate that the calculated imposition of water deficit can effectively decouple vegetative growth from reproductive quality. In grapevine, for instance, moderate RDI has been shown to promote significant water savings and to control excessive vegetative vigor while maintaining yield and enhancing fruit physicochemical quality, including increases in soluble solids, phenolic compounds, and antioxidant capacity [21,22,23]. These quality improvements are largely attributed to the stimulation of secondary metabolite biosynthesis as part of plant defense and acclimation mechanisms under moderate stress. Moreover, classical studies have demonstrated that RDI markedly improves water use efficiency (WUE) without major penalties in productivity [24]. Accordingly, the contribution of recent work is not the “discovery” of beneficial stress per se, but rather the refinement of its mechanistic basis, thresholds, and crop-specific implementation, reinforcing stress as a context-dependent factor that can be leveraged as a strategic management tool for adding value to the final agricultural product, particularly in water-limited environments.

Consequently, an effective “systemic shift” emerges from the convergence of agronomic management and genetic potential. The application of priming (preconditioning) techniques has proven crucial for inducing tolerance mechanisms by preparing plant metabolism for a more robust defense response [25,26]. Simultaneously, the exploration of genotypes such as ancestral wheat varieties reveals a highly favorable genetic architecture to drought tolerance, often surpassing that of modern cultivars and opening new avenues for crop improvement [27]. This integration ultimately leads to a new physiological equilibrium, optimizing the trade-off between defense and growth [28].

Salinity imposes a particularly stringent constraint on SPAC functioning because it combines an immediate osmotic phase—which reduces the soil solution water potential and can mimic “physiological drought”—with a slower but often more damaging ionic phase driven by the accumulation of Na^+^ and Cl^−^ in sensitive tissues. This dual nature disrupts water uptake and hydraulic conductance while progressively impairing cellular homeostasis through nutrient antagonism (e.g., reduced K^+^ retention and altered Ca^2+^ signaling), membrane destabilization, and enzyme inhibition [29,30]. At the whole-plant scale, these processes amplify stomatal limitation and depress carbon assimilation, increasing the likelihood of excess excitation energy in photosynthetic tissues and thereby intensifying ROS production. When antioxidant and osmoprotective systems are insufficient, oxidative injury (lipid peroxidation, protein oxidation, and loss of membrane integrity) and osmotic dehydration become mutually reinforcing, accelerating senescence and limiting growth and yield [31,32]. Therefore, salinity tolerance emerges as an integrated trait requiring coordinated ion exclusion and compartmentalization, osmotic adjustment via compatible solutes, and efficient redox buffering—features that can be targeted through genotype selection and management practices such as irrigation water quality control, leaching strategies, and rootstock-mediated ion homeostasis [33,34,35].

## 4. Conclusions and Future Perspectives

Overall, the papers in the present Special Issue highlight the potential of integrating molecular insights with agronomic and ecological tools to enhance plant resilience against adverse impacts resulting from climate change. It is increasingly important that plant tolerance is not viewed in isolation. To enhance our understanding of these interactions, more dedicated studies into how molecular signals (like ABA and GABA) operate under field conditions [8,9], and how ecological buffers (like mosses) can be integrated into agricultural systems [16], are needed. We hope this collection inspires further investigation into the “hidden mechanisms” that bridge the gap between gene expression and ecosystem sustainability.
